# Total Lip Reconstruction with Tendinofasciocutaneous Radial Forearm Flap

**DOI:** 10.1155/2014/219728

**Published:** 2014-02-04

**Authors:** Eldad Silberstein, Yuval Krieger, Yaron Shoham, Ofer Arnon, Amiram Sagi, Alexander Bogdanov-Berezovsky

**Affiliations:** Department of Plastic Surgery, Soroka University Medical Center, Ben-Gurion University of the Negev, P.O. Box 151, Beersheba 84101, Israel

## Abstract

*Introduction.* Squamous cell carcinoma is a common tumour of lower lip. Small defects created by surgical resection may be readily reconstructed by linear closure or with local flaps. However, large tumours resection often results with microstomia and oral incompetence, drooling, and speech incomprehension. The goal of this study is to describe our experience with composite free radial forearm-palmaris longus tendon flap for total or near total lower lip reconstruction. *Patients and Methods.* This procedure was used in 5 patients with 80–100% lip defect resulting from Squamous cell carcinoma. Patients' age ranged from 46 to 82 years. They are three male patients and two female. In 3 cases chin skin was reconstructed as well and in one case a 5 cm segment of mandible was reconstructed using radius bone. In one case where palmaris longus was missing hemi-flexor carpi radialis tendon was used instead. All patients tolerated the procedure well. *Results.* All flaps totally survived. No patient suffered from drooling. All patients regained normal diet and normal speech. Cosmetic result was fair to good in all patients accept one. *Conclusion.* We conclude that tendino-fasciocutaneous radial forearm flap for total lower lip reconstruction is safe. Functional and aesthetic result approaches reconstructive goals.

## 1. Introduction

Squamous cell carcinoma is a common tumor of lower lip that requires radical surgery. Small defects created by surgical resection may be readily reconstructed by linear closure or with local flaps. However, large defects with large defects involving over 60% of lip local flaps previously described by Gillies and Millard [1], Karapandzic [2], Webster et al. [3], Nakajima et al. [4], and others often results with microstomia and oral incompetence resulting in drooling and speech incomprehension [5, 6]. Microstomia may result with impaired oral hygiene and inability to use dentures. When tumor involves adjacent facial regions such as cheek and chin local and regional flaps are even less reliable in providing enough tissue and therefor living the patient with severe deformity. The reconstructive goal is to restore mucosal lining and external skin coverage and restoring oral competence so as to achieve good oral food intake without drooling, understandable speech, opportunity for good oral hygiene care, and acceptable aesthetics [7]. Sakai et al. [7] first described the use of a composite radial forearm along with palmaris longus tendon to reconstruct large lower lip defects. The folded skin island provides mucosal lining and skin coverage while the palmaris longus tendon is sutured to the modiolus on both ends and serves as a suspension sling. The goal of this study is to describe our experience with composite free radial forearm-palmaris longus tendon flap.

## 2. Patients and Methods

Between May 2005 and July 2008 we used tendino-fasciocutaneous radial forearm flap for total or near total lower lip reconstruction in five patients. Age of patients ranged from 46 to 82 years. They are three male patients and two females. All tumors were T3 or T4 and tumour resection resulted with lip defect of 80–100%. Patients 2–4 received postoperative radiation therapy. Follow-up time ranged between three and six years. The details of patients' tumours are summarized in Table 1.

### 2.1. Operative Technique

**Figure 1:**
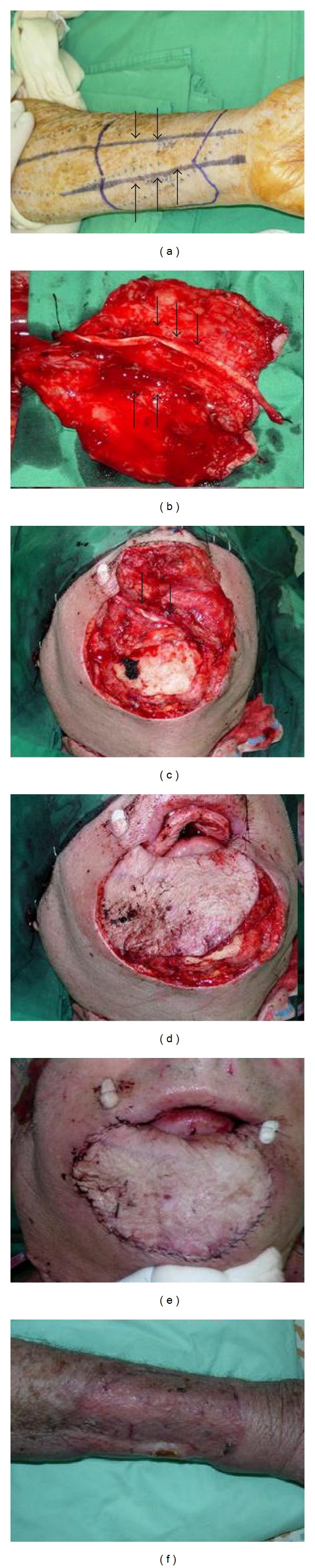
Surgical technique. (a) Marking of radial artery (two arrows) and palmaris longus tendon (three arrows), (b) Flap elevated showing vascular pedicle (thin arrows) and palmaris longus tendon (thick arrows) attached to the under surface of the flap by thin peritendon. (c) Part of the flap is sutured to the oral mucosa for lining and the tendon (Arrows) is secured to the Modioulus, Buccinator muscle. (d) The skin paddle is folded over the tendon (e) and suturing it in place. (f) Donor site is covered with split thickness skin graft.

**Figure 2:**
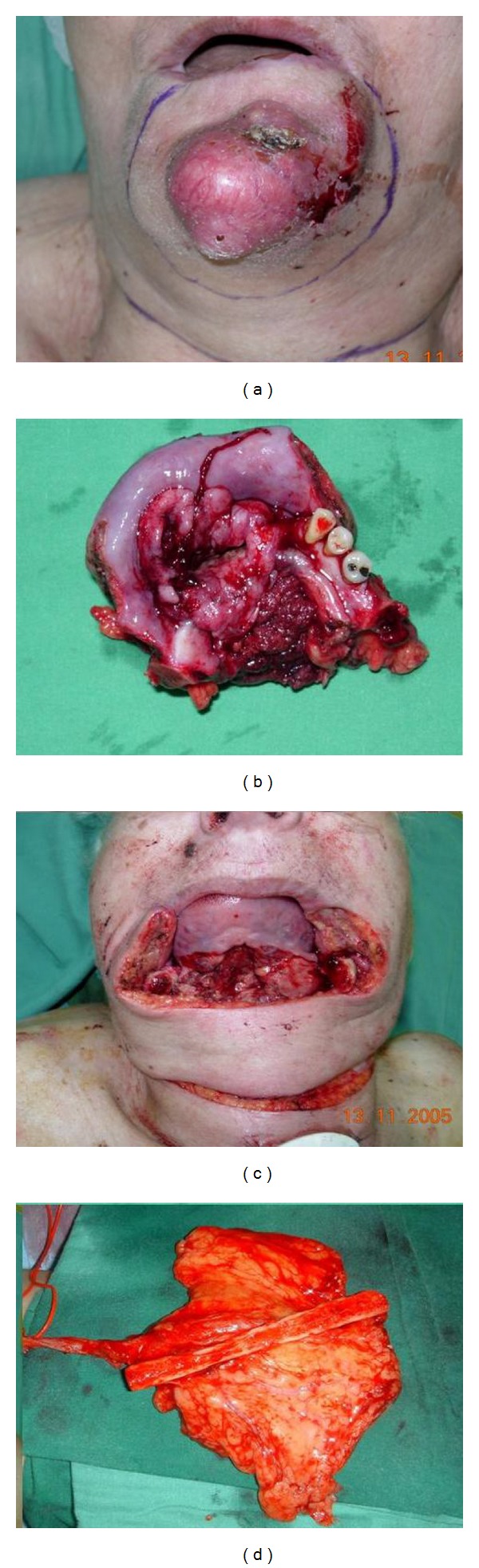
Patient number 2. (a) Tumor involving lower lip, chin, anterior segment of Mandible and flour of mouth. (b) Tumor resected. (c) Resulting defect (d) Flap elevated with a segment of radius bone attached thru periosteum to thevascular pedicle.

**Figure 3:**
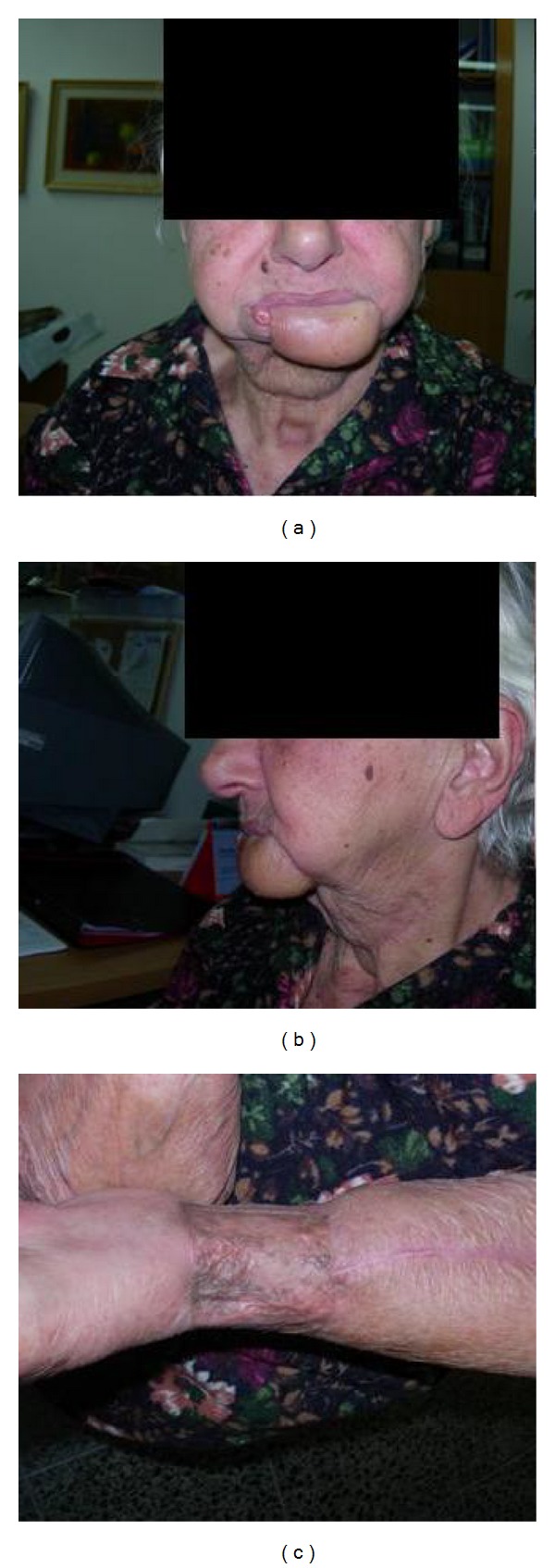
Patient number 2: result: (a) anterior view. (b) side view, and (c) donor site.

All patients underwent radical resection of tumor combined with supraomohyoid neck dissection flowed by immediate reconstruction. Lip defects ranged 80–100%. In all cases the defect was reconstructed using a tendino-fasciocutaneous radial forearm free flap. Skin paddle was designed according to mucosal and skin defects (Figure 1(a)). Three patients needed reconstruction of chin skin as well as lip and one patient needed reconstruction of anterior floor of mouth. The flap was raised in a usual manner except for special care to keep the palmaris longus tendon with the flap (Figure 1(b)). This part of the dissection is delicate since the tendon is attached to the under surface of the skin paddle by peritendon only. The pedicle was tunnelled to the neck and microvascular anastomosis was performed. The tendon was anchored on both sides to the modiolus and buccinator muscle and the skin paddle was sutured to the oral mucosa (Figure 1(c)). The flap was then folded over the tendon and sutured in place (Figures 1(d) and 1(e)). The donor site was covered with skin graft (Figure 1(f)). In one case palmaris longus muscle and tendon were absent and half of flexor carpi radialis tendon was harvested and used in a similar manner as a tendon graft. In one case (Figures 2 and 3) the resection included a 5 cm segment of anterior mandible that was reconstructed using a vascularised segment of radius with the flap. The radius bone segment is a wedge of about one-third of bone circumference or cross-sectional area. No plating of the remaining bone was done and the patient was casted for two weeks. The two mandible segments and the bone flap were plated using a reconstruction plate.

## 3. Results

**Figure 4:**
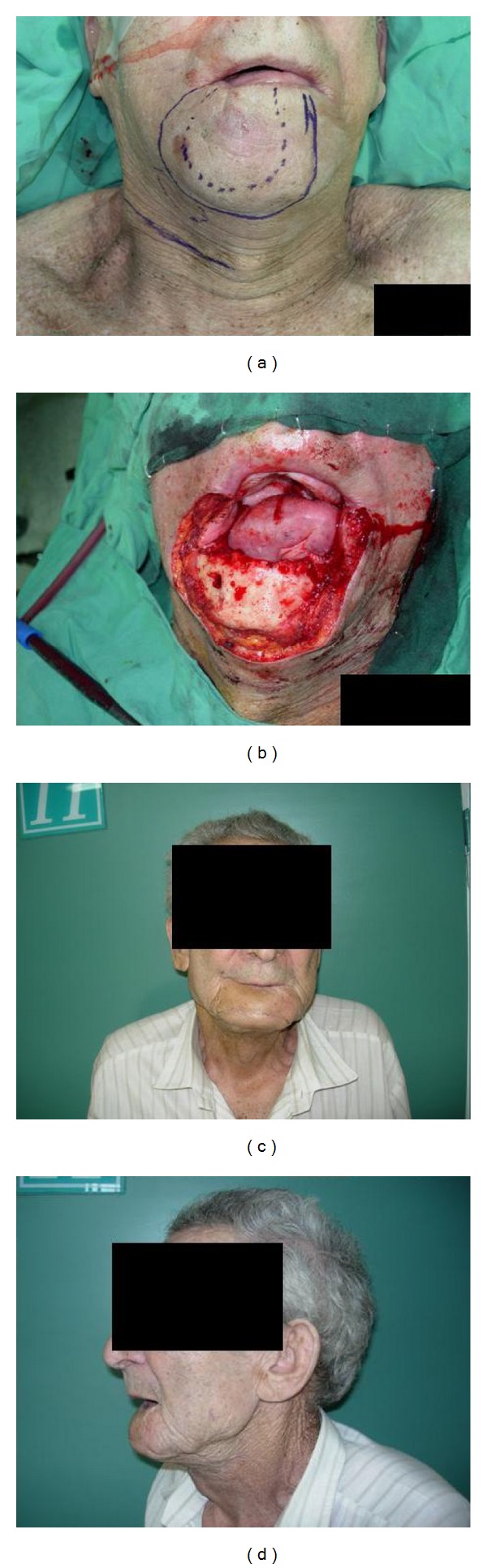
Patient number 1. (a) Preoperative marking. Tumor border (dashed line) planed resection (continuous line). (b) Intraoperative defect showing 80% thru en thru lower lip defect and chin defect with exposed mandible. ((c), (d)) Postoperative result.

**Figure 5:**
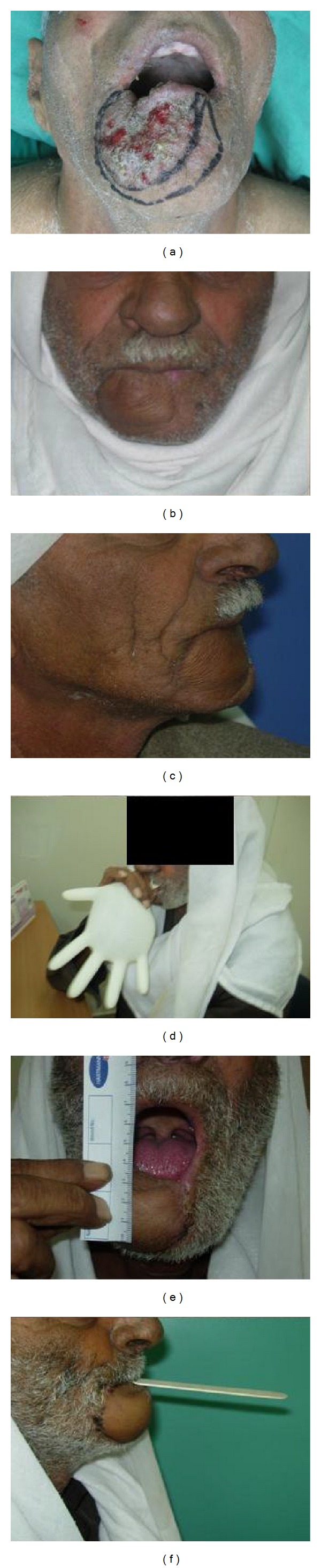
Patient number 5: (a) preoperative tumor resection outlined, (b) Postoperative anterior view, and (c) lateral view. Lip function: (d) patient is blowing a rubber glove. (e) Mouth wide open. (f) Patient is holding a wooden mouth opener with his lips only showing good lip competence.

Patients' outcome is summarized in Table 2. All patients survived the procedure and were discharged home back to their previous day-to-day activities. All flaps totally survived. All patients regained normal diet and good oral competence. Aesthetic result was usually good or excellent (Figures 4 and 5). One patient with a composite reconstruction of lower lip chin mandible and mouth floor resulted with a less than acceptable result. Although we estimate that secondary revision may have resulted with significant improvement, the patient did not consent to this. Four patients regained normal speech and one patient reported minor speech and communication difficulties. None of the patients suffered microstomia or problems with oral hygiene maintenance.  Figure 5 shows good mouth opening and good lips competence with the patient blowing a rubber glove and holding a wooden mouth opener with his lips only. No significant donor site morbidity was obtained (Figures 1(f) and 3(c)).

## 4. Discussion

Total or near total lower lip reconstruction remains a challenge to the reconstructive surgeon. Goals of the reconstruction procedure are to restore patients oral competence, speech, and diet as well as to permit oral hygiene and as normal as possible appearance [6–8]. Local flaps often fail to achieve these goals and require numerous revision procedures [5]. Also, regional skin flaps from the neck may suffer from questionable blood supply after neck dissection and radiation prior to surgery. Several methods of suspension procedures have been introduced in the literature. We find the method described by Sakai et al. [7] and modified by others [5, 6, 8] very useful in these difficult cases. In this paper we describe our experience with total lip reconstruction using the tendino-fasciocutaneous radial forearm flap. We find this procedure to be safe and to achieve most reconstructive goals. In one of our patients we found the palmaris longus to be absent; in this case we used half of the flexor carpi radialis tendon as a suspension sling. To the best of our knowledge this is the first report of that technique. We would like to point out to the readers that about 15% of patients are lacking the palmaris longus [9, 10]. Therefore it is important to bear in mind the option of using hemi-FCR tendon in this case. In one case we incorporated also a piece of radius to reconstruct a segment of mandible. We have not seen such a report before. Incorporation of bone into the flap makes planning harvesting and insetting of the flap somewhat difficult and limited. We were reluctant to attempt to segment the bone flap since it is relatively small and thin so we were apprehend to shatter it. This obviously limits one's ability to create good chin contour due to the limited bone bulk and safety with ostectomy of the partial thikness bone. Radial forearm flap is not our flap of choice for mandible reconstruction; however in some elderly patients with significant peripheral vascular disease when the use of fibula flap is not feasible due to occlusion of peroneal vessels, it may serve as a second option in spite of its limitations.

## Figures and Tables

**Table 1:** Patient and tumor characteristics.

Patient	Age (y)/sex	Tumor stage	% of lip defect	Adjacent tissue involvement and resection	Remarks
1	72/M	T4N0M0	80	Chin	Recurrent tumor, radiation therapy failure
2	82/F	T4N0M0	80	Chin, mandible, and mouth floor	
3	56/M	T3N0M0	90		
4	46/F	T3N0M0	100		
5	58/M	T4N0M0	80	Chin	

**Table 2:** Patients Outcome.

Patient	Postoperative complication	Flap survival	Diet	Static lip suspension	Oral competence	Speech	Aesthetic acceptance	Remarks
1	No	100%	Normal	Good	Adequate	Normal	Excellent	Vermilion reconstruction with mucosal graft.
2	No	100%	Normal	Good	Adequate	Minor difficulties	Less then acceptable	Combined with mandible reconstruction by radius bone in the flap. Flap to bulky, patient refused debulking procedure.
3	Wound infection: incision and drainage	100%	Normal	Good	Adequate	Normal	Good	
4	Venous insufficiency: Leeches therapy	100%	Normal	Good	Adequate	Normal	Acceptable	Debulking surgery 6 months postoperatively.
5	No	100%	Normal	Good	Adequate	Normal	Excellent	No palmaris longus. Hemi-flexor carpi radialis tendon was used for suspension.
